# Free Electron–Plasmon Coupling Strength and Near-Field Retrieval through Electron Energy-Dependent Cathodoluminescence Spectroscopy

**DOI:** 10.1021/acsnano.3c12972

**Published:** 2024-05-14

**Authors:** Evelijn Akerboom, Valerio Di Giulio, Nick J. Schilder, F. Javier García de Abajo, Albert Polman

**Affiliations:** † Center for Nanophotonics, NWO-Institute AMOLF, Science Park 104, 1098 XG Amsterdam, The Netherlands; ‡ ICFO-Institut de Ciencies Fotoniques, The Barcelona Institute of Science and Technology, Castelldefels, 08860 Barcelona, Spain; § Gleb Wataghin Physics Institute, University of Campinas, Campinas 13083-859, Brazil; ∥ ICREA-Institució Catalana de Recerca i Estudis Avançats, Passeig Lluís Companys 23, 08010 Barcelona, Spain

**Keywords:** cathodoluminescence spectroscopy, strong light−matter interaction, free electron−light interactions, plasmons, confined optical modes, near-field distributions

## Abstract

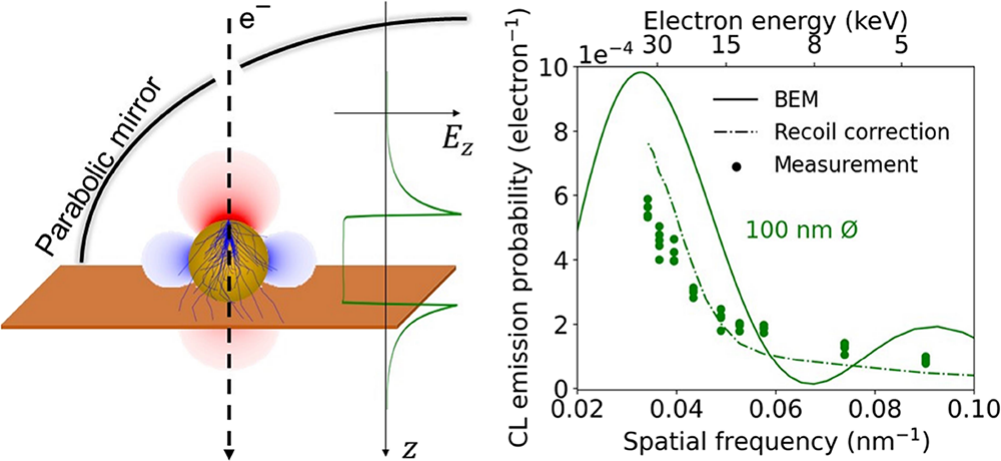

Tightly confined optical near fields in plasmonic nanostructures play a pivotal role in important applications ranging from optical sensing to light harvesting. Energetic electrons are ideally suited to probing optical near fields by collecting the resulting cathodoluminescence (CL) light emission. Intriguingly, the CL intensity is determined by the near-field profile along the electron propagation direction, but the retrieval of such field from measurements has remained elusive. Furthermore, the conditions for optimum electron near-field coupling in plasmonic systems are critically dependent on such field and remain experimentally unexplored. In this work, we use electron energy-dependent CL spectroscopy to study the tightly confined dipolar mode in plasmonic gold nanoparticles. By systematically studying gold nanoparticles with diameters in the range of 20–100 nm and electron energies from 4 to 30 keV, we determine how the coupling between swift electrons and the optical near fields depends on the energy of the incoming electron. The strongest coupling is achieved when the electron speed equals the mode phase velocity, meeting the so-called phase-matching condition. In aloof experiments, the measured data are well reproduced by electromagnetic simulations, which explain that larger particles and faster electrons favor a stronger electron near-field coupling. For penetrating electron trajectories, scattering at the particle produces severe corrections of the trajectory that defy existing theories based on the assumption of nonrecoil condition. Therefore, we develop a first-order recoil correction model that allows us to account for inelastic electron scattering, rendering better agreement with measured data. Finally, we consider the albedo of the particles and find that, to approach unity coupling, a highly confined electric field and very slow electrons are needed, both representing experimental challenges. Our findings explain how to reach unity-order coupling between free electrons and confined excitations, helping us understand fundamental aspects of light–matter interaction at the nanoscale.

Understanding the nanoscale distribution of light fields in the optical spectral range is of great importance in many technologies. For example, in photovoltaics, nanoscale near-field scattering determines the light trapping efficiency;^1,2^ in photochemistry, the strength of surface modes determines the efficiency of generating chemical fuels;^3,4^ and in integrated optics, near-field coupling determines the propagation and coupling of optical signals. Noble-metal and dielectric nanostructures support strong plasmonic and Mie resonances that can be geometrically tailored to better trap light or more effectively capture heat. Their optical near fields are tightly confined to the nanoparticle surface within a typical range of 10–50 nm.^5^ To design these nanostructures, it is crucial to have a method to probe the electric near-field distributions at the nanoscale. However, probing the amplitude and phase of the electric near field is proven to be challenging with optical techniques because it displays small features, far below the diffraction limit.

In recent years, high-energy (1–200 keV) electron beams (e-beams) have emerged as probes of optical near fields.^6−8^ Energetic electrons act as a broadband excitation source, which, due to their small de Broglie wavelength (39–2.5 pm for 1–200 keV electrons) and the numerical aperture of electron microscopes, can be spatially positioned with far better resolution than light.^5^ Although the absorption or emission of a net number of photons by an electron is kinematically forbidden in free space, an energetic electron passing near or through a polarizable structure can efficiently couple to the near-field components of electromagnetic modes, which, in turn, can radiate to the far field. The interaction of the electron with the induced optical near field can be sensitively probed by measuring the electron energy in electron energy-loss spectroscopy (EELS),^9^ while cathodoluminescence (CL) spectroscopy relies on the study of the emitted radiation, which is collected in the far field.^10^ The inverse process (far-field photons illuminating a structure and coupling to the electron through the induced near fields) has emerged as an exciting approach to gain control over electron light–matter interaction in the so-called photon-induced near-field electron microscopy (PINEM) technique, which leverages the near field created by scattering of an intense external laser at a nanostructure to dramatically enhance the interaction strength.^11^ Such strong coupling then reshapes the electron wave function into a superposition state observed with an electron spectrometer as a set of energy-loss and energy-gain sidebands corresponding to the emission or absorption of one or more photons. In this context, control of the optical near-field distribution provides a way to tailor the electron wave function.^12,13^

In all three techniques, EELS, CL, and PINEM, the electron near-field interaction strength for an electron moving along the *z* direction is determined by the spatial distribution of the *z* component of the electric field *E*_*z*_(*z*) that is probed. Yet, so far, a detailed experimental study of the electric near field along the electron trajectory and its associated coupling strength has remained missing. EELS, CL, and PINEM investigations of near fields have focused mostly on acquiring maps of the near-field strength in the *x*–*y* plane (i.e., perpendicular to the incident electron direction), integrating the electron near-field interactions along the *z*-axis. For example, EELS and CL measurements showed *x*–*y* maps of the resonant modes of plasmonic nanotriangles and nanowires with ultrahigh spatial resolution.^14,15^ Likewise, CL and PINEM measurements have revealed *x*–*y* maps of the transverse-electric modes in photonic crystal cavities.^16,17^ In 3D reconstruction techniques, like electron tomography, information about the third dimension can be obtained by rotating the sample and subsequent numerical processing,^18^ as exemplified by Nicoletti et al.,^19^ who visualized the 3D distribution of localized surface plasmon resonances in gold nanocubes using EELS, and also by Atre et al.,^20^ who demonstrated the 3D and spectral reconstruction of nanocrescents using CL. However, the reconstruction of the actual electric field is not a trivial task and has only been tackled partially with these different techniques. The near field has three spatial components, each of them complex for each optical frequency. In addition, the electron only couples to the field component along the e-beam direction.

In this article, we leverage the electron-energy dependence of CL spectra to experimentally study tightly confined plasmonic optical near fields in gold nanoparticles. In particular, we investigate spherical plasmonic Au nanoparticles with diameters in the 20–100 nm range. We address the question of how the coupling strength depends on electron energy, the induced near-field distribution, and the e-beam position (impact parameter). From this, we derive previously inaccessible spatial information on the induced electric near field along the *z* direction (i.e., the e-beam direction). We study the coupling strength between gold nanoparticles and electrons with energies in the range of 4–30 keV in two different configurations: aloof excitation in which the electron passes close to the particle (grazing with respect to the surface); and penetrating excitation, where the electron passes through the center of the particle. In agreement with theory, we find that faster electrons couple better to optical near fields described by lower spatial frequencies. Additionally, we introduce a first-order recoil correction to the coupling strength for penetrating electrons, accounting for the strong effect of elastic and inelastic electron scattering inside the particle. Finally, by correcting for the plasmonic scattering efficiency of the nanoparticles, we extract absolute values for the electron-to-near-field coupling. Overall, the data provide insight into the electron-energy dependence and optimization of electron–plasmon coupling, the near-field distribution, and the subsequent CL emission. Our data are relevant to tailor electron light–matter interactions in CL, EELS, and PINEM experiments, especially when specific conditions of strong coupling are sought.

## Theory

Fundamentally, EELS, CL, and PINEM signals are governed by the coupling dynamics between individual free electrons and the electric field carried by the moving electron (EELS, CL) or supplied by an external laser pulse (PINEM). In the nonrecoil approximation (i.e., assuming that the electron velocity vector remains unchanged during the time of interaction), the CL emission probability (Γ_CL_) for a single mode excited by an electron moving at a constant speed *v* along the *z* direction is proportional to the work done by the electron on the optical modes of the system along its trajectory,^7,21,22^
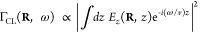
1with *E*_*z*_ denoting the *z* component of the induced electric near field, ***R*** = (*x*, *y*) the impact parameter of the electron, and ω the resonance frequency of the excited mode (ω = 2π*c*/λ, where λ is the associated light wavelength). From a classical perspective, this expression can be understood as the effect of alternating acceleration and deceleration of the electron as it traverses the induced electric near field, with a net deceleration resulting in energy loss and subsequent emission of radiation. Equation 1 shows how Γ_CL_ directly represents the Fourier transform of *E*_*z*_ at a spatial frequency *q* = ω/*v*. This implies that a near-field distribution that is phase-matched with the passing electron (i.e., fields characterized by a central wave vector centered near ω/*v*) leads to the strongest CL intensity. Consequently, slow electrons (high *q*) induce CL mostly for near-field distributions with large spatial frequencies, corresponding to small spatial features, while fast electrons couple best to near-field components with small spatial frequencies. So far, no CL experiments have systematically studied these trends.

**Figure 1:**
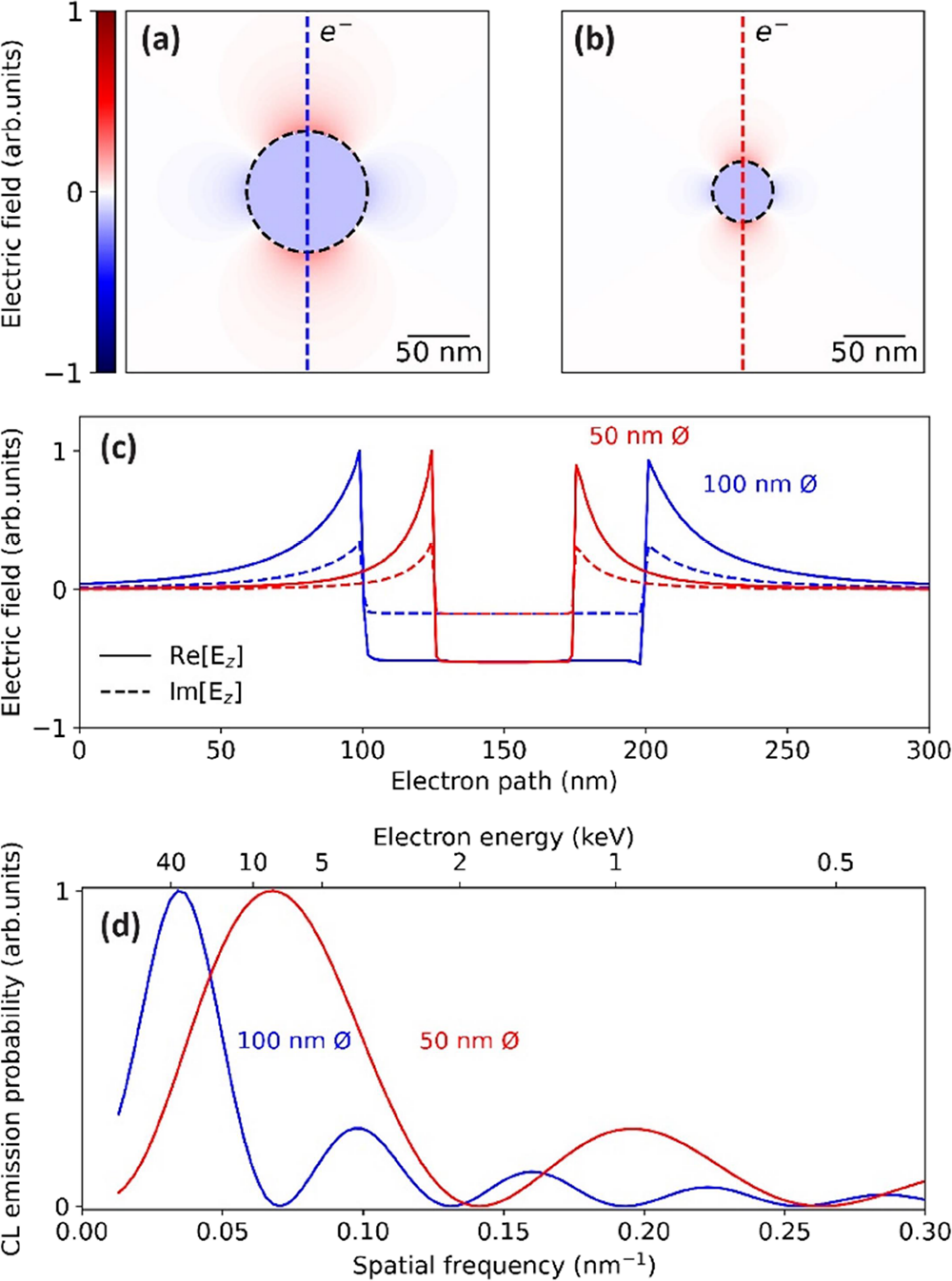
(a, b) Electric field of a *z*-oriented plasmonic dipole induced by a plane wave at 530 nm wavelength in (a) a 100 nm and (b) a 50 nm diameter gold spherical particle, calculated using BEM.^23,25^ (c) Real (solid) and imaginary (dashed) parts of the *z* component of the electric field along the *z*-axis for *x*,*y* = 0 in particles of 100 nm (blue) and 50 nm (red) diameter. (d) Squared modulus of the spatial Fourier transform of the complex *z* component of the electric field in panel (c), which is proportional to the CL emission probability.

We start by examining the theory for the excitation of a dipolar plasmonic mode in a gold spherical nanoparticle. Figure 1 shows fixed-time snapshots of the real part of *E*_*z*_ for a dipolar mode in gold nanospheres of 100 (a) and 50 nm (b) diameter placed in vacuum and excited by a light plane wave at the resonance wavelength of 530 nm. The electric fields are computed through a robust numerical solution of Maxwell’s equations based on the boundary element method (BEM).^23,24^ We first consider the excitation of this dipolar mode by a fast electron penetrating the particle along the central particle axis (dashed line). The dipolar field profile that is induced by the electron, and acting back on it along the trajectory, is shown in Figure 1c for two particle sizes under consideration. Both cases show a homogeneous field inside the particle and a strong, highly confined near field at the edge of the particle. Following eq 1, we take the Fourier transform of the *E*_*z*_ profiles in Figure 1c to calculate the CL emission probability as a function of the spatial frequency *q* carried by the electron (Figure 1d). The corresponding electron energy is shown on the top axis. For resonances in the optical range, we corroborate that fast electrons (30–40 keV) induce CL mostly for near-field distributions with small spatial frequencies, corresponding to the larger features in the 100 nm diameter particle. In contrast, slower electrons couple best with near-field components with higher spatial frequencies (small features). Figure 1d also reveals that, to achieve a maximum of CL emission for a dipolar mode, the spatial electron frequency *q* should match the excited mode, such that *q*∼ (2*n* + 1)π/*D*, with *n* an integer and *D* the diameter of the particle. For a 100-nm particle, the dipolar mode is optimally excited with an electron carrying a spatial frequency *q* = 0.03 nm^–1^ (30 keV for λ = 530 nm), while *q* = 0.07 nm^–1^ (8 keV) is best for a 50 nm particle. We stress that these electron energies are all accessible in a standard scanning electron microscope (SEM).

To experimentally study the strength of the coupling between electrons and plasmonic nanoparticles, we perform CL measurements in a SEM operating at acceleration voltages of 4–30 keV. The SEM is equipped with a CL collection system consisting of a half-parabolic mirror and an optical spectrometer. The mirror is positioned between the electron column and the sample plane. Using a microactuation stage, the focal point of the mirror is aligned with the e-beam and the emitted light is directed through a vacuum port onto an optical spectrometer. To minimize the influence of the substrate, single-crystalline Au nanospheres are drop-casted on a 15 nm thick Si_3_N_4_ membrane and cleaned using an oxygen plasma to remove the PEG carboxyl ligands (see Methods section). We measured the CL spectrum for particles of 20–100 nm diameter in two configurations: a penetrating e-beam configuration, described above, and an aloof configuration in which the electron is passing near the particle at a distance of 5 ± 2.5 nm from its surface. For every particle diameter, the electron energy is decreased from 30 to 4 keV, and every measurement is repeated five times on an unexposed particle. Additionally, we measured the angular emission profile of the CL signal and observed the characteristic emission for a dipole mode (data not shown). To complement our data, we use BEM simulations and the analytical dipole model.

## Results and Discussion

### Aloof Configuration

**Figure 2:**
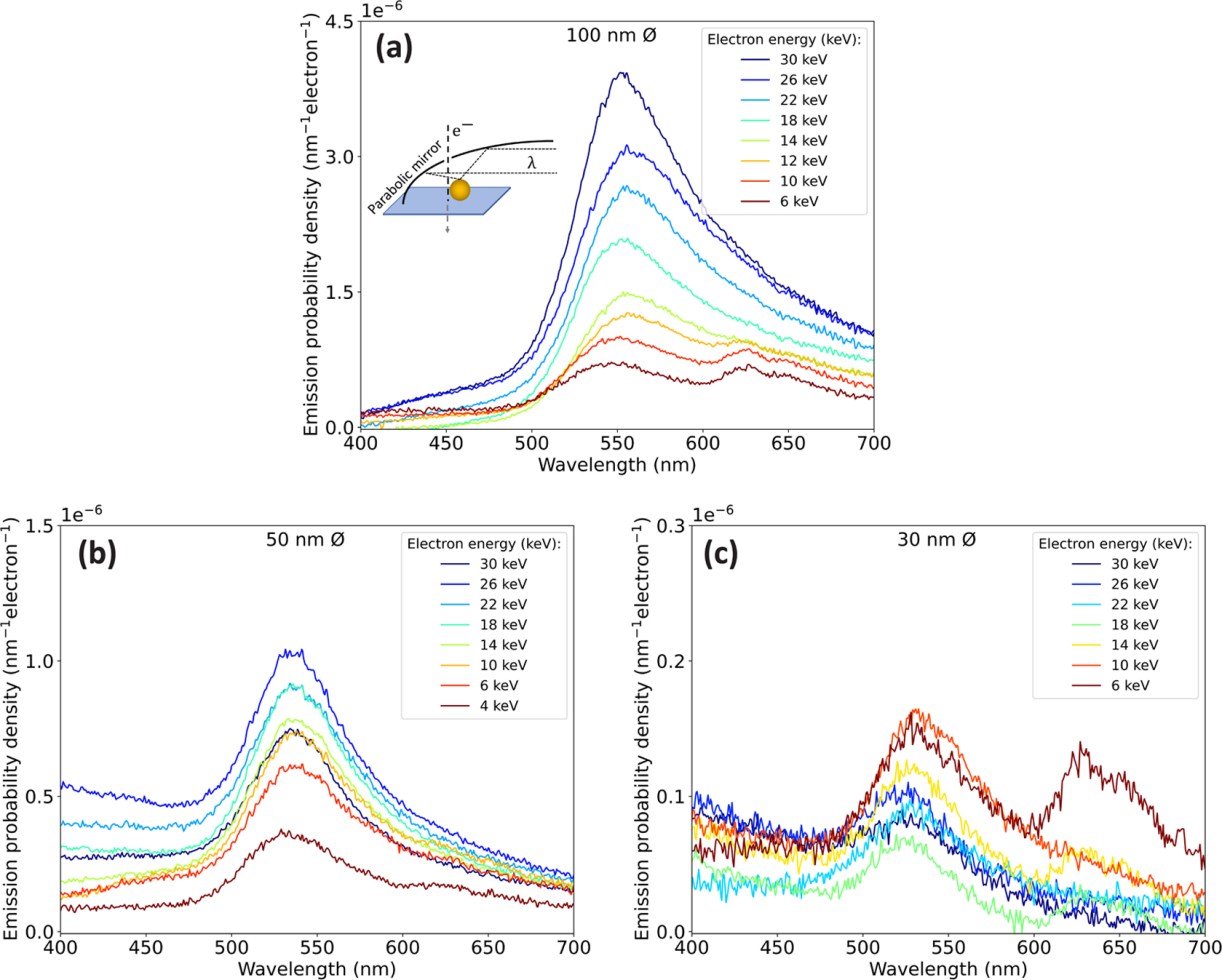
Measured CL emission probability for gold spheres of (a) 100, (b) 50, and (c) 30 nm diameter, excited by 4–30 keV electrons (dark red to blue, respectively), which are passing close to the particle at a distance of 5 ± 2.5 nm.

Figure 2 shows the measured CL spectra for aloof excitation of gold particles with diameters of 100 (a), 50 (b), and 30 (c) nm for electron energies in the 6–30 keV range. We refer to Supporting Information Figure S1 for details on the spread in the measurements and comparison to BEM simulations. All spectra exhibit a strong dipolar resonance at an emission wavelength of 530 nm, varying in intensity with electron energy. Furthermore, the spectrum first shows a slight redshift with decreasing electron energy and then a blueshift. This is due to the small irregularities in particle shape that cause the peak to shift. While the intensity for the largest particle monotonically increases with electron energy up to 30 keV, we observe a maximum for the 50 nm diameter particle at 26 keV electron energy. For the smallest particle, no clear trend can be observed because of the large relative error in the measured spectra (note the difference in vertical scale for the three different particle sizes). In some measurements for slow electrons and small particles, we observe a spectral feature around 650 nm superimposed on the plasmon spectrum. We ascribe this feature, which does not depend on particle size, to emission from carbon deposited as a result of exposure to the e-beam.

**Figure 3:**
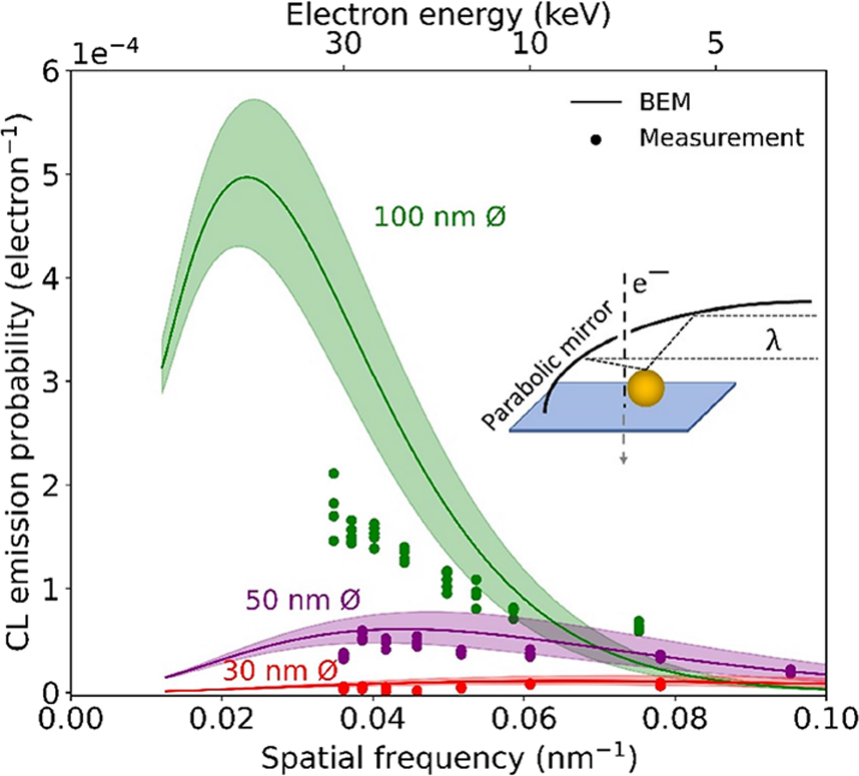
Measured (dots) and simulated (solid) CL emission probability for aloof excitation of gold nanospheres with a diameter of 100 (green), 50 (purple), and 30 nm (red). Experimental data points are obtained by integrating the emission probability from Figure 2 over a bandwidth of 60 nm around the peak wavelength in each measured spectrum. The bandwidths around the solid lines show the uncertainty of the impact parameter, which is estimated as *b* = 5 ± 2.5 nm. Figure S2 shows the same data on a logarithmic scale to reveal details in the low-signal data.

To compare the trends in Figure 2 to theory, we integrate the plasmon peak over a 60 nm bandwidth around the peak wavelength. We plot the resulting emission probability versus the spatial frequency *q* carried by the electron for the three particle sizes in Figure 3 together with BEM simulations for the same geometries. Overall, the decreasing trend of CL efficiency with spatial frequency is well represented by the data for the 100 nm diameter particles. However, if we compare the absolute values measured in the experiments with the BEM simulations, we see that, for the 100 nm diameter particles, this is half of the value that BEM predicts. We attribute this discrepancy to the interaction with the substrate, which is not incorporated in the BEM simulations. A substrate can cause a large part of the light to be channeled into the substrate, reducing the intensity that is collected by the parabolic mirror. In the 50 nm diameter particles, a maximum coupling strength is found at *q* = 0.04 nm^–1^, representing the fact that these particles display a more tightly confined near field with less prominent low-*q* components. No clear trend is observable for the 30 nm diameter particles.

### Penetrating Configuration

**Figure 4:**
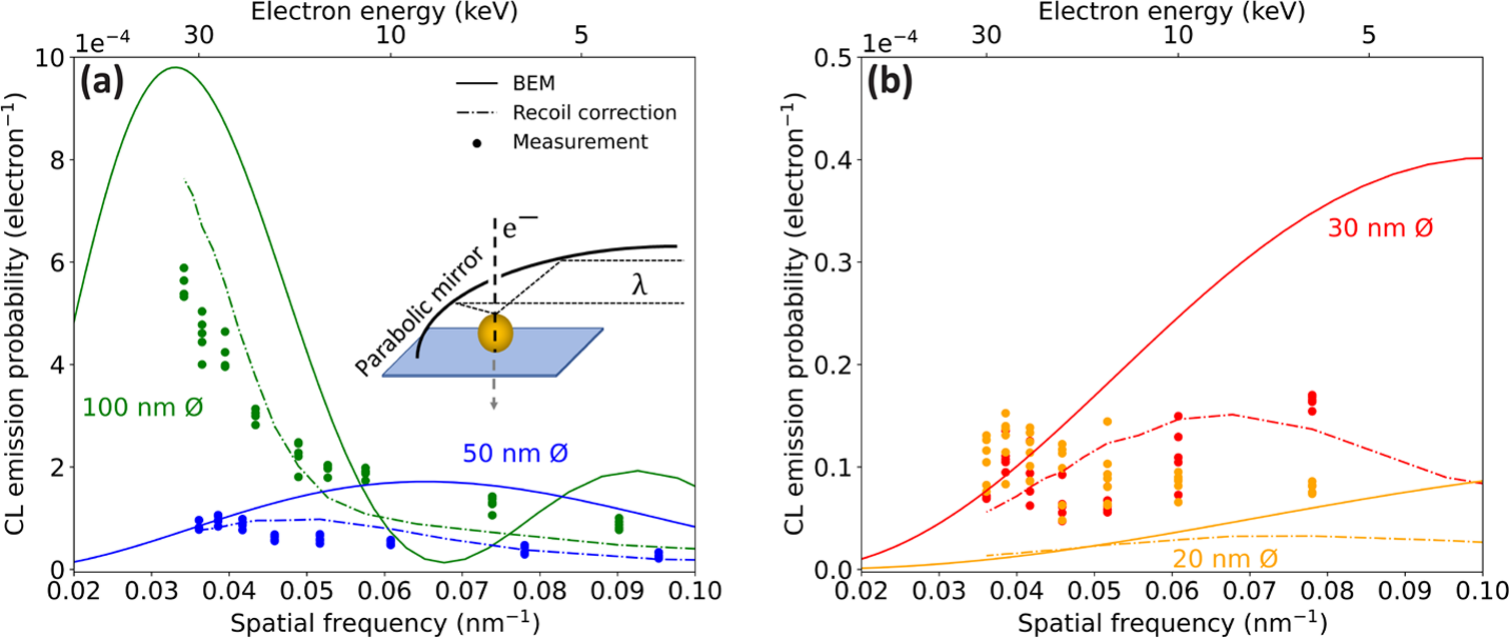
Measured (dots) and simulated (solid) CL emission probability for electrons passing through the center of gold nanospheres with a diameter of 100 (green) and 50 nm (purple) in (a), and 30 (red) and 20 nm (blue) in (b). The emission probability is integrated over a 60 nm bandwidth around the peak wavelength. The dashed curves show the CL emission probability calculated using a recoil correction on eq 1, taking into account the penetration depth of the electron, normalized to the analytical nonrecoil calculation (see Methods section).

Next, we investigate the coupling strength of electrons to plasmonic modes for excitation along the particle axis. Figure 4a,b shows the CL measurements versus electron energy for particles of 100 (green) and 50 nm (purple) diameter, and 30 (red) and 20 nm (blue), respectively (see Supporting Information Figure S3 for the spectral data). BEM simulations show the same behavior predicted from the analytical description above, with maximum CL emission probability where the spatial frequency matches the excited mode such that *q* = π/*D*.

However, in contrast to the data for aloof excitation in Figure 3, we observe significant differences between experiments and simulations: first, for the largest particle, BEM simulations show an emission probability that is roughly twice larger than the one measured for high electron energies; and second, for the 50-nm diameter particles, the recorded spectra show a reduced coupling with increasing *q*, while the simulations predict an increase up to *q* = 0.07 nm^–1^. Most notably, the upward trend in the calculations for the 30-nm diameter particles is not seen in the experiments. We argue again that, for the smallest particles (Figure 4b), the data does not reveal a clear trend due to the higher signal-to-noise ratio.

BEM simulations are based on the assumption that an electron maintains its velocity and momentum during the entire time of interaction (nonrecoil approximation). Here, this assumption proves to be invalid because the electrons have a high probability of undergoing elastic and inelastic collisions within the nanoparticle. We ascribe the discrepancy between the measured CL emission probability and the numerical predictions to the effect of such scattering. As a first-order correction to the model to take recoil effects into account, we modify the integration boundaries in eq 1 to include only the range of the electron trajectory inside the nanoparticle. We use Monte Carlo simulations^26^ to obtain statistics on the penetration depth for a given electron energy. We then evaluate the integral in eq 1 and normalize it to the maximum coupling efficiency found for the simulated nonrecoil scenario. The data derived using this recoil-corrected model are plotted in Figure 4 (dotted lines) for the four particle diameters under consideration.

The recoil calculation shows a lower emission probability due to the termination of the integral, bringing the model closer to the data for the particles of 100, 50, and 30 nm diameter. While the BEM simulation shows a vanishing emission probability at *q* = 0.07 nm^–1^ for the 100-nm particle because the near field does not have a component at this spatial frequency, the recoil-corrected model produces a finite probability, consistent with the measurement. This is a direct result of electron recoil, associated with the fact that the electron trajectory ends inside the particle and, therefore, does not probe the full Fourier integral through the entire particle.

This analysis shows the importance of accounting for electron recoil effects in quantifying absolute CL emission probabilities. Furthermore, it provides insight into the best conditions for coupling free electrons and nanoparticle plasmons. In brief, for the larger particles, faster electrons (energies around 30 keV) match best to the large spatial features, while for the smaller particles, the electron velocity must be carefully matched to the spatial frequency. In addition, recoil, which corrects the distribution of such frequency, needs to be considered to obtain optimal coupling.

While the analysis of CL emission allows us to estimate the coupling between an electron and free electromagnetic radiation mediated by the confined excitations supported in the sample, the direct electron-mode coupling is of particular relevance in the study of electron–light correlations.^27^ To estimate this quantity, we consider the plasmonic scattering albedo that is determined by the balance between radiative and nonradiative plasmon decay processes. The plasmon radiative efficiency ranges from 1 to 30% for particles of 30–100 nm diameter. Correcting for the albedo (see Methods section), we can derive the electron–plasmon coupling strength in our experiments.

**Figure 5:**
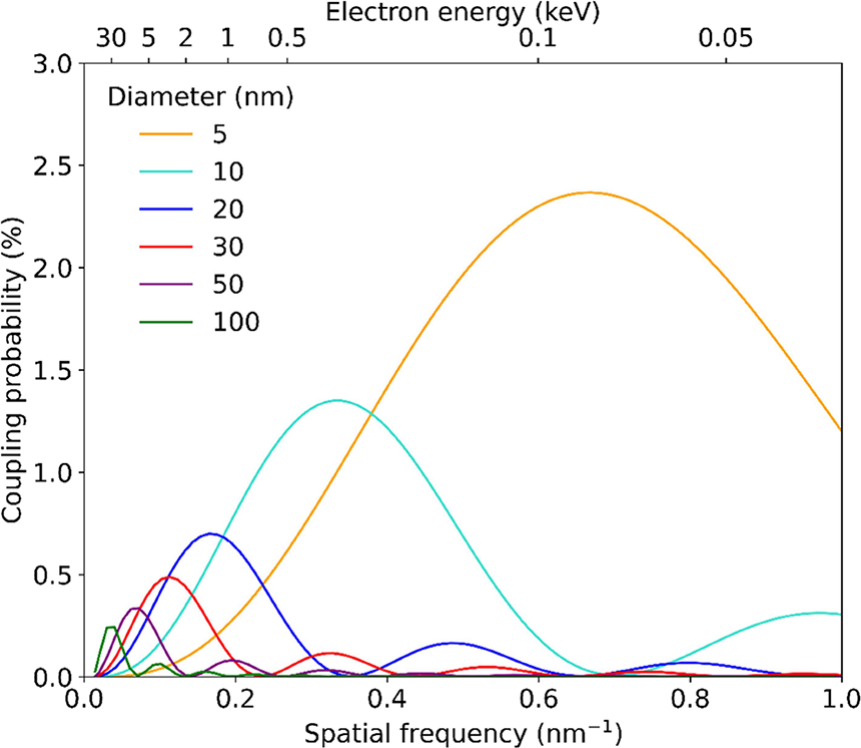
Simulated coupling probability between the electron and the near field induced in spherical gold nanoparticles with a diameter of 100 (green), 50 (purple), 30 (red), 20 (blue), 10 (turquoise), and 5 nm (orange) for penetrating trajectories (passing near the particle center), calculated using BEM with a fwhm of the e-beam width of 0.1 nm and corrected for the plasmon scattering albedo (see Methods section).

Figure 5 shows the CL emission probability from a BEM simulation corrected for the albedo of gold spherical nanoparticles of 5–100 nm diameter. To study the fundamental limit of electron–plasmon coupling, we use an e-beam full-width at half-maximum (fwhm) of 0.1 nm (see Supporting Information Figure S5 for an analogous calculation with a fwhm of 5 nm). This graph directly represents the electron–plasmon coupling strength. By using the results shown in Figure 5, we can now compare the absolute coupling strength between particles of different sizes. The spatial frequency for which a maximum coupling is observed increases for smaller particles, in agreement with the Fourier analysis described above. Furthermore, we observe that the peak in coupling strength increases with decreasing particle diameter. Quantitatively, we find the highest coupling strength of 2.5% for a 5-nm diameter particle at an electron energy of 100 eV. Further increased coupling can be achieved for even more strongly confined near fields (<5 nm) and very slow electrons (<100 eV). Such experiments would be challenging in a SEM and inspire geometries where electrons are accelerated in specially tailored vacuum geometries using a strong electric field.

To further investigate the scaling properties in electron-driven resonant excitations, we performed in parallel a theoretical study of electron near-field coupling for a range of resonant plasmonic, dielectric, and polaritonic excitations.^28^ In particular, we show that a few percent coupling strength can be achieved for gold nanospheres with a diameter of 5 nm excited by low-energy electrons (1 keV). This can be further improved to unity-order coupling for ultrasmall (few nm diameter) resonant particles at low electron (<100 eV) energies. This is due to phase matching (see above) combined with the higher overall coupling strength at lower electron velocity. The insights from these calculations, combined with the experimental study in the present paper, inspire practical geometries for the use of electrons as sources for spectroscopy at the nanoscale.

## Conclusions

In conclusion, we have systematically examined the coupling strength between swift electrons and the dipolar mode in gold plasmonic nanospheres for both aloof and penetrating configurations. Under aloof excitation, our measurements confirm the validity of the nonrecoil approximation model in which a maximum of CL emission is observed when the electron spatial frequency (*q* = ω/*v*) matches that of the near field associated with the excited mode (here dipolar). In contrast, for penetrating e-beams, the nonrecoil approximation breaks down because of the large changes produced in the electron trajectory due to elastic and inelastic scattering inside the nanoparticle, including substantial deflection and deceleration. We present a modified model, taking these recoil effects into account, and find better agreement with the measured data.

To investigate the absolute electron-to-near-field coupling strength, we corrected the simulated data for the effect of radiative losses by dividing the CL emission probability by the albedo of the particle and extrapolated this to very small particle sizes. The extrapolated data show a maximum coupling strength of 2.5% for strongly confined near fields (<5 nm) and low-energy electrons (100 eV).

These results lie at the edge of the capabilities of conventional SEMs and inspire geometries for high-efficiency CL generation from ultrasmall optical near fields excited by using low-energy (<100 eV) electrons. Our work not only guides us toward strong-coupling conditions for electron–plasmon interaction, but it also provides fundamental insight into the control and optimization of optical excitations in nanostructures, with a potential future in nanoscale optoelectronic circuits for a wide range of applications.

## Methods

### Sample Preparation

Gold colloidal particles were purchased from nanoComposix (San Diego), with diameters of 20, 30, 50, and 100 nm. The particles had PEG carboxyl ligands and were delivered as an aqueous solution with a 0.05 mg/mL concentration of gold. The particles were diluted 1–100 in demi water and sonicated for 2 min. Before drop-casting 2 μL from the suspension onto a 15 nm thick Si_3_N_4_ grid (Ted Pella), the surface was made hydrophilic using a UV-zone cleaner (BioForce UV/Ozone ProCleaner) for 10 min. After drop casting, the sample was cleaned with an Oxygen plasma for 2 min using an Oxford Instruments Plasmalab 80 Plus tool.

### CL Measurements

CL measurements were performed in an FEI Quanta FEG 650 SEM (Thermo Fisher Scientific Inc., MA, USA) equipped with a Schottky electron source. The CL collection system was composed of a parabolic mirror between the sample and the pole piece, which collected the emitted light from the top hemisphere. The light was directed into an optical detection system (SPARC Spectral, DELMIC BV, The Netherlands).^29^ The measurements were done with an e-beam current of 230–1000 pA, depending on the electron energy, with an acquisition time of 0.2 s for 100 nm diameter, 1 s for 50 and 30 nm diameter, and 2 s for 20 nm diameter particles. In the aloof configuration, a 2D map of the entire particle was collected using a pixel width of 5 nm. By analyzing the secondary-electron images, the pixels at 5 nm from the edge were found with an accuracy of 2.5 nm. To correct for the CL background signal, dark counts were subtracted from the data for the penetrating geometry, while CL from the Si_3_N_4_ support membrane was subtracted from the aloof data. The system response was calibrated using the measured transition radiation (TR) of single-crystalline aluminum. The TR was benchmarked to an analytical expression^8^ and used to obtain the absolute CL probabilities.

### BEM Simulation

Numerical calculations were performed using the BEM^25^ as implemented in the MNPBEM17 Matlab toolbox.^23,24^ Spherical nanoparticles were used and parametrized by 144 triangular face elements, with optical constants for gold taken from Olmon et al.^30^ For the computation of the induced dipole field inside the gold nanoparticle (Figure 1), a plane-wave excitation was introduced at a wavelength of 530 nm, incident along the *x* axis with polarization along the *z*-axis. The induced dipole corresponds to the excited mode upon electron excitation along the *z*-axis. For calculations of the CL emission probabilities, built-in functions were used, assuming a fwhm of the e-beam waist of 5 nm.

### Recoil Correction

To incorporate the effect of electron scattering while traversing through a gold spherical nanoparticle, eq 1 was corrected to truncate the integral at the penetrating depth of the electron:
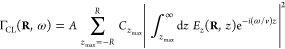
2with *A* the proportionality factor, *z*_max_ the electron penetration depth, and *C*_*z*_max__ a weighting factor describing contributions from electrons for a given penetration depth, as derived from Casino simulations (see below). The proportionality factor *A* is taken equal to the one for the nonrecoil picture and is used to normalize the analytical expression to data from BEM calculations.

### Monte Carlo Simulations

A CASINOv2.5 Monte Carlo program^26^ was used to obtain the distribution of electron paths in the electron cascade and determine *C*_*z*_max__ for every electron energy and penetrating depth *z*_max_ considering electrons incident on a planar gold slab (density of 19.3 g/cm^3^). The maximum depth that was reached before the electron was either backscattered or absorbed was assigned to *z*_max_. If the electron passed further than the diameter of the particle, it was set to be transmitted, with *z*_max_ = −∞. See Supporting Information Figure S4 for the electron penetration statistics.

### Albedo Calculation

To account for the optical radiative efficiency of the gold nanoparticle, we compared the analytical formulation for EELS and CL emission probabilities. We used the analytical expressions obtained for an induced electrical dipole by a grazing electron, given by^31^
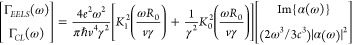
3The EELS signal represents the total energy loss of the electron along the trajectory while the CL signal corresponds to the excitation fraction that is radiated toward the far field. We use this as an approximation to obtain the CL radiative efficiency (η) for penetrating trajectories, by dividing the CL emission probability by the EELS probability, which results in
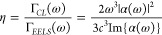
4In the electrostatic limit (*a* ≪ λ), the polarizability tensor, α, is given by^32^
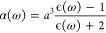
5with *a* the radius of the particle and ϵ the dielectric constant of the particle. However, for the actual sizes of our particles, we need to use Mie theory to incorporate retardation corrections in the description of the particle polarizability, so we set
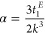
where *t*_1_^*E*^ is the dipolar electric Mie scattering coefficient.^8^

## Supporting Information Available

Plots of spectra acquired from gold nanoparticles in aloof (Figure S1) and penetrating (Figure S3) configurations, along with the corresponding BEM simulated spectra; the data from Figure 3 on a logarithmic scale to reveal details of the low-signal data (Figure S2); the penetration depth statistics for 4 and 10 keV electrons impacting on a planar gold surface (Figure S4); and the albedo correction for all particle sizes for a fwhm of the e-beam waist of 5 nm (Figure S5) (PDF)
