# Supplementary material for: Free Electron–Plasmon Coupling Strength and Near-Field Retrieval through Electron Energy-Dependent Cathodoluminescence Spectroscopy
Source: ACS Nano. 2024 May 14;18(21):13560–7. doi: 10.1021/acsnano.3c12972 (PMC11140833; doi:10.1021/acsnano.3c12972)
Supplement: Supplementary file 1 — nn3c12972_si_001.pdf [file nn3c12972_si_001.pdf]

Free Electron-Plasmon Coupling Strength and Near-Field Retrieval through Electron Energy-Dependent Cathodoluminescence Spectroscopy

Evelijn Akerboom*[1], Valerio Di Giulio[2], Nick J. Schilder[1,3], F. Javier García de Abajo[2,4], and Albert Polman[1]

* Corresponding authors: e.akerboom@amolf.nl

[1]Center for Nanophotonics, NWO-Institute AMOLF, Science Park 104, 1098 XG Amsterdam, the Netherlands

[2]ICFO-Institut de Ciencies Fotoniques, The Barcelona Institute of Science and Technology, 08860 Castelldefels, Barcelona, Spain

[3]Gleb Wataghin Physics Institute, University of Campinas, 13083-859 Campinas, SP, Brazil

[4]ICREA-Institució Catalana de Recerca i Estudis Avançats, Passeig Lluís Companys 23, 08010 Barcelona, Spain

Supporting information

S1: CL spectra in the aloof configuration

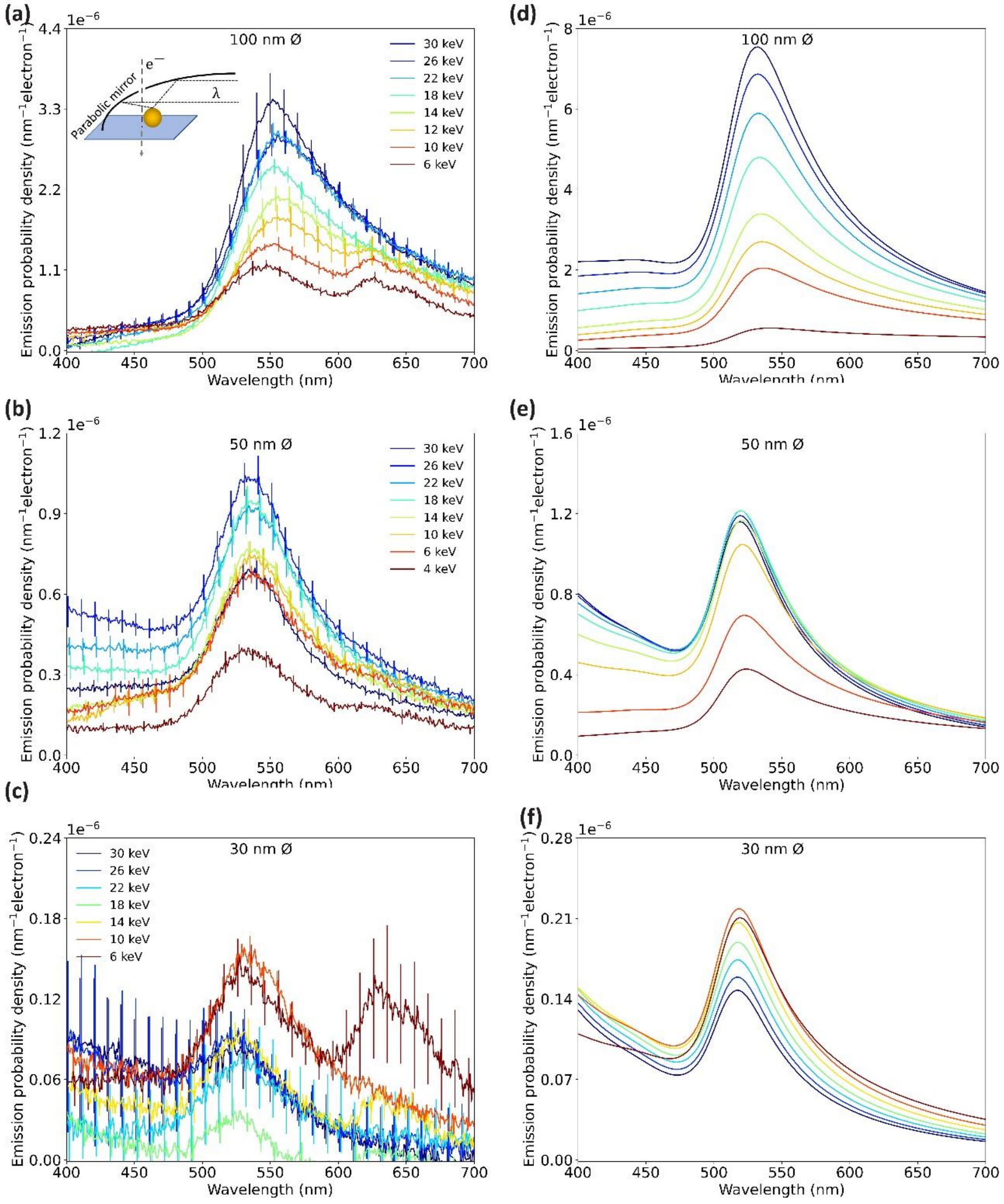

Figure S1. Measured (a-c) and simulated (d-f) CL spectra for particles of 100 – 30 nm diameter excited with a 6 – 30 keV e-beam in the aloof configuration (electrons that graze the particle surface). The error bars in the measured data show the variance for different measurements from spheres of the same size.

S2: CL emission probability for the dipolar mode excited in the aloof configuration

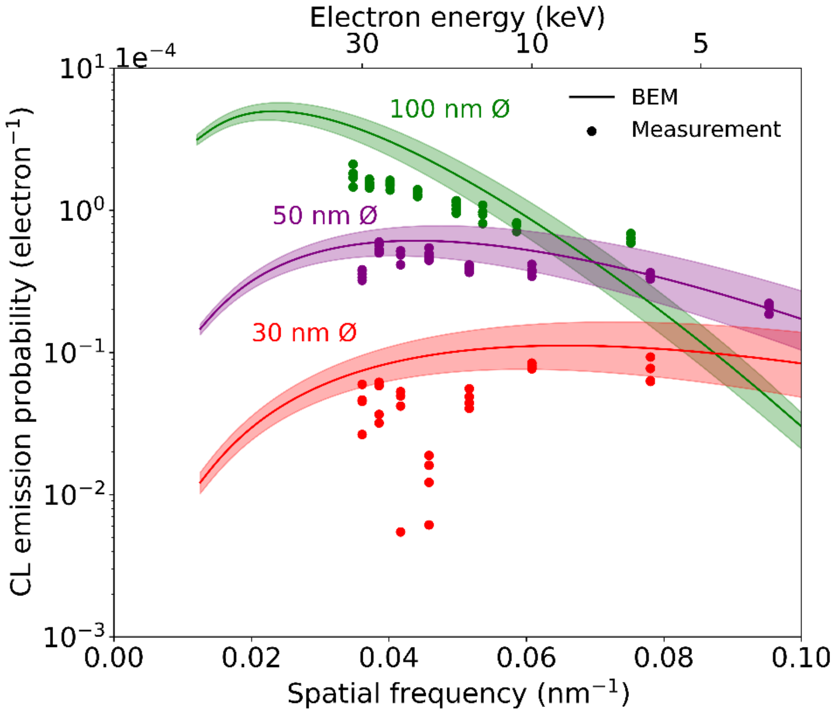

Figure 2. Measured (dots) and simulated (solid) CL emission probability for aloof excitation of gold nanospheres with a diameter of 100 nm (green), 50 nm (blue), and 30 nm (red). Experimental data points are obtained by integrating the emission probability spectra from Figure 2 over a bandwidth of 60 nm around the peak wavelength. The bandwidth around the solid curves shows the effect of uncertainty in the impact parameter, which is estimated as $b = 5 \pm 2.5$ nm.

S3: CL spectra for penetrating electron beams

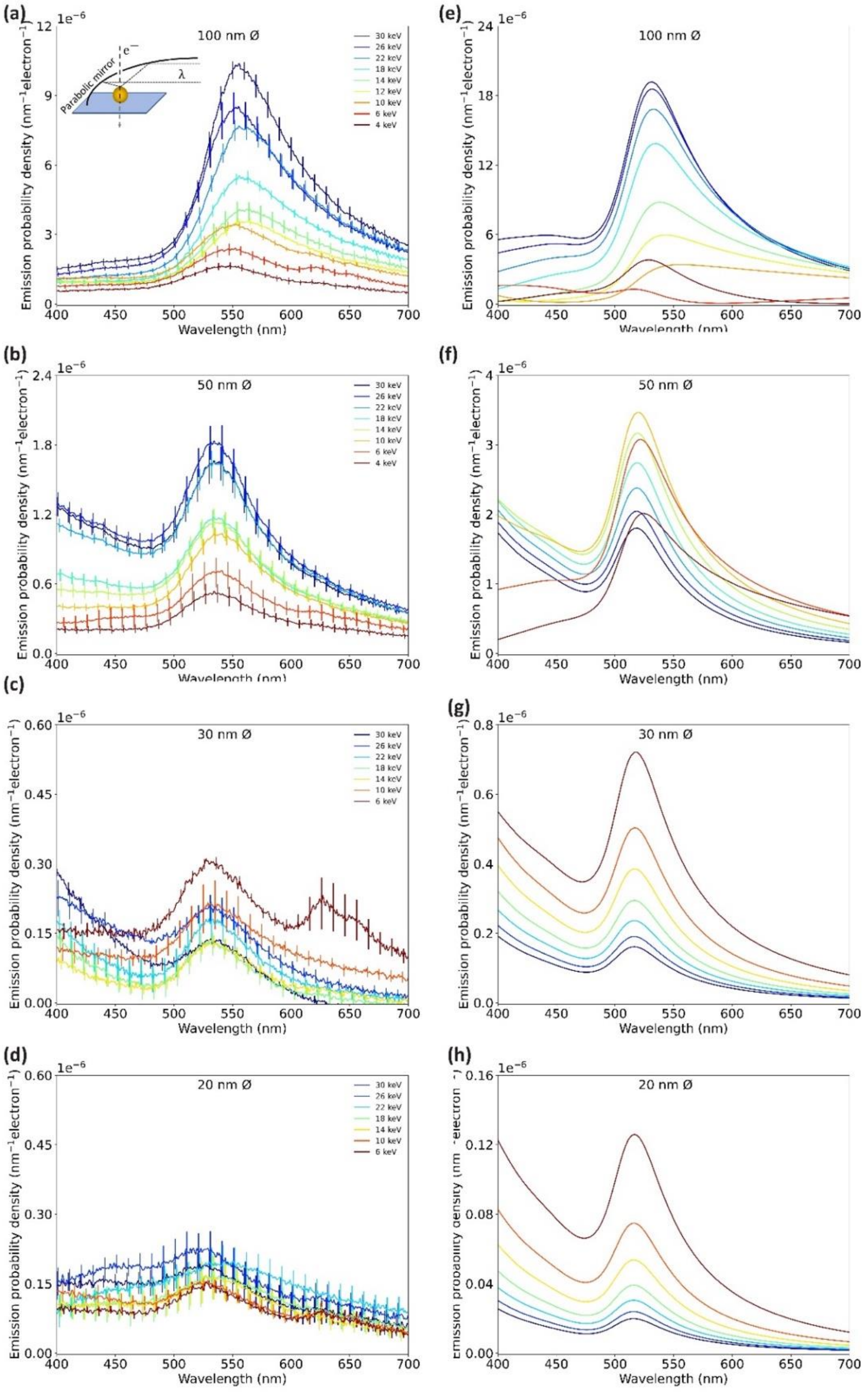

Figure S3. Measured (a-d) and simulated (e-h) CL spectra for particles of 100 – 20 nm diameter excited by penetrating 6 – 30 keV electrons aimed at the particle center. Error bars in the measured data show the variance for different measurements from spheres of the same nominal size.

S4: Monte-Carlo simulations for electrons traversing a planar gold surface

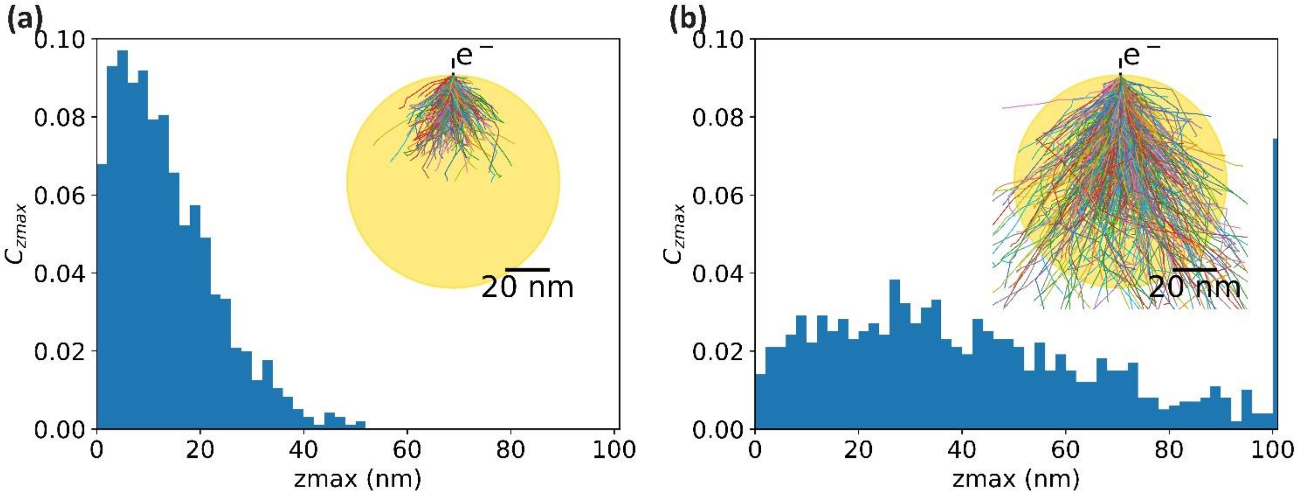

Figure S4. Monte-Carlo statistics of the penetration depth (z_{max}) of 4 keV (a) and a 10 keV (b) electrons penetrating a planar gold surface. The insets show the dispersion of electron trajectories during propagation through the gold material. Contours of a spherical Au particle (100 nm diameter, yellow) are shown for reference.

S5: Albedo correction to the excitation efficiency

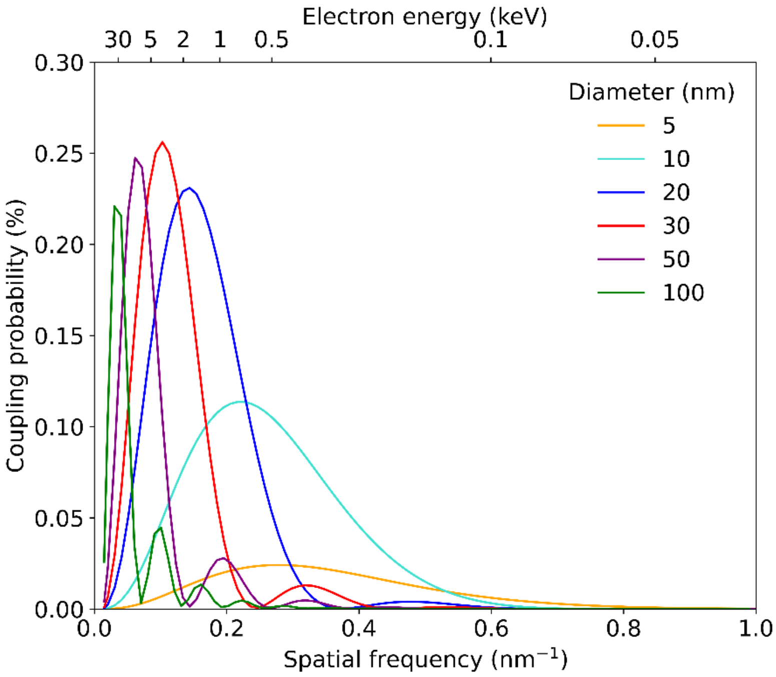

Figure S5. Simulated coupling probability between the electron and the induced near field in spherical gold nanoparticles with a diameter of 100 nm (green), 50 nm (purple), 30 nm (red), 20 nm (blue), 10 nm (turquoise), and 5 nm (orange) for penetrating electron trajectories, as calculated with BEM for an electron waist of 5 nm FWHM, corrected for the plasmon scattering albedo (see Methods).
